# Nuclear RNA Regulation by XRN2 and XTBD Family Proteins

**DOI:** 10.1247/csf.21041

**Published:** 2021-09-03

**Authors:** Ilkin Aygün, Takashi S. Miki

**Affiliations:** 1 Department of Developmental Biology, Institute of Bioorganic Chemistry, Polish Academy of Sciences, Poznań, Poland

**Keywords:** RNA regulation, XRN2, XTBD, ribosome biogenesis, subcellular localization

## Abstract

XRN2 is a 5'-to-3' exoribonuclease that is predominantly localized in the nucleus. By degrading or trimming various classes of RNA, XRN2 contributes to essential processes in gene expression such as transcription termination and ribosome biogenesis. Despite limited substrate specificity *in vitro*, XRN2 targets a specific subset of RNA by interacting with other proteins in cells. Here we review the functions of proteins that have an evolutionarily conserved XRN2-binding domain, XTBD. These proteins modulate activity of XRN2 by stabilizing it, controlling its subcellular localization or recruiting it to specific RNA targets, and thereby impact on various cellular processes.

## XRN2 and XTBD

Ribonucleases control cellular RNA levels by degrading mature RNA or by processing RNA precursors for maturation, thereby supporting gene regulation. They also contribute to the maintenance of cells’ health by eliminating useless or potentially harmful RNA such as aberrant RNA species and the remnants of RNA biogenesis (Wolin and Maquat, 2019). XRN2 is an evolutionarily conserved 5'-to-3' exoribonuclease that is distributed in two compartments of the nucleus: the nucleoplasm and the nucleolus. Since original identification as ribonucleic acid trafficking protein 1 (Rat1) in *Saccharomyces cerevisiae* (Amberg *et al.*, 1992), a number of studies have revealed its diverse functions (Nagarajan *et al.*, 2013; Miki and Großhans, 2013; Kurihara, 2017) such as tRNA quality control (Chernyakov *et al.*, 2008; Payea *et al.*, 2020), heat-induced translational repression (Watanabe *et al.*, 2013, 2014), regulation of microRNA turnover (Chatterjee and Grosshans, 2009), elimination of the remnants of microRNA biogenesis (Kurihara *et al.*, 2012), and restriction of viral replication (Li *et al.*, 2015). Among those, transcription termination and ribosome biogenesis have been particularly studied. Roles of XRN2 in transcription termination of RNA polymerase II (Pol II) have been described in yeast (Kim *et al.*, 2004), humans (West *et al.*, 2004) and *Caenorhabditis elegans (C. elegans)* (Miki *et al.*, 2017). Following endonucleolytic cleavage of a nascent transcript at a polyadenylation site by the 3'-end processing machinery that is composed of the endonuclease Ysh1/CPSF73 and other factors (Kumar *et al.*, 2019), XRN2 degrades the downstream transcript until it catches up with Pol II to dissociate it from DNA. The similar mechanism functions at transcription start sites in mammals, where XRN2, in conjunction with decapping factors and the termination factor TTF2, can terminate transcription by Pol II (Brannan *et al.*, 2012). XRN2 has been shown to play critical roles in processing of precursor ribosomal RNAs (pre-rRNAs) in several species (Amberg *et al.*, 1992; Petfalski *et al.*, 1998; Fang *et al.*, 2005; Zakrzewska-Placzek *et al.*, 2010; Couvillion *et al.*, 2012; Preti *et al.*, 2013). Following endonucleolytic cleavage of pre-rRNA, XRN2 trims the 5' extension to form a precise 5' end of mature rRNA.

**Fig. 1:**
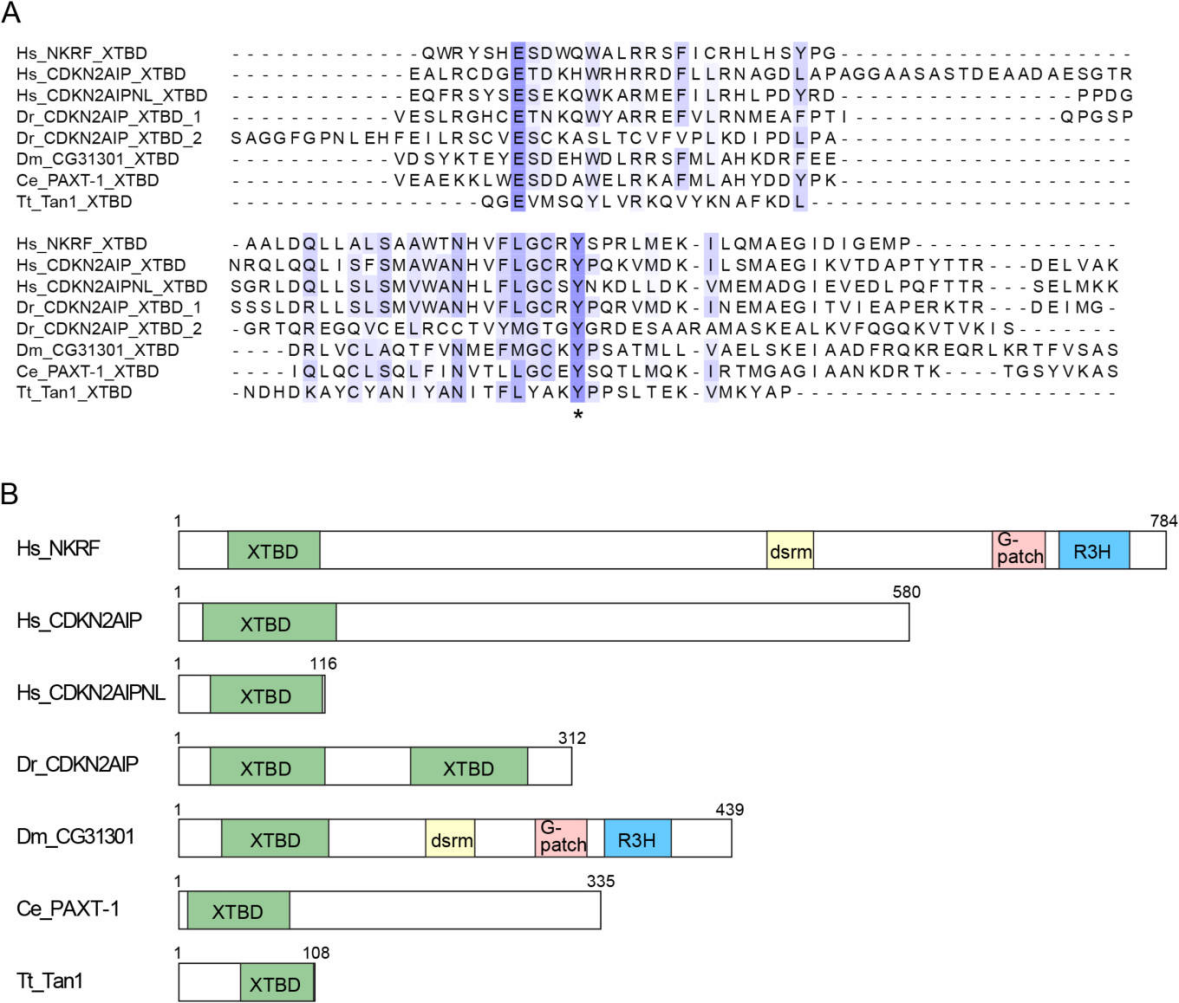
XTBD family proteins. (A) XTBDs of *Homo sapiens* (Hs) NKRF (39–111 aa), CDKN2AIP (19–124 aa), and CDKN2AIPNL (25–113 aa), *Danio rerio* (Dr) CDKN2AIP (24–114 aa, 184–276 aa), *Drosophila melanogaster* (Dm) CG31301 (34–118 aa), *Caenorhabditis elegans* (Ce) PAXT-1 (7–87 aa), and *Tetrahymena thermophila* (Tt) Tan1 (49–106 aa) are aligned by Clustal Omega (https://www.ebi.ac.uk/Tools/msa/clustalo/) and visualized by Jalview (https://www.jalview.org/), based on the Pfam database. The most frequent amino acids are in blue and similar amino acids in light blue, based on BLOSUM62. A highly conserved tyrosine required for interaction with XRN2 is marked with an asterisk. (B) Domain architectures of XTBD family proteins based on the Pfam database. Amino acid numbers relative to the first methionine are shown. XTBD: XRN-Two Binding Domain; dsrm: Double-stranded RNA binding motif; G-patch: G-patch domain; R3H: R3H domain. Amino acid numbers relative to the first methionine are shown.

Since XRN2 degrades 5'-monophosphorylated single-stranded RNA without apparent sequence specificity (Stevens and Poole, 1995), its recruitment to specific subcellular compartments or RNA targets by interacting partners is important to exert cellular functions. In the exploration of such factors, Miki *et al.* identified a *bona fide* XRN2-binding domain (XTBD), previously known as the domain of unknown function 3469 (DUF3469), in the *C. elegans* protein Partner of XRN-Two-1 (PAXT-1) (Miki *et al.*, 2014a). They further demonstrated that two XTBD-containing proteins, NF-κB repressing factor (NKRF) and CDKN2A interacting protein (CDKN2AIP), bind to XRN2 in humans. A follow-up study by the team solved the structure of an XTBD-XRN2 complex (Richter *et al.*, 2016). The researchers identified binding interface residues that create hydrophobic and polar bonds between the two proteins, of which mutation of a highly conserved tyrosine in the XTBD of PAXT-1 completely abrogated the interaction (Fig. 1A).

According to the Pfam database, >800 XTBD family proteins have been found across >350 species in metazoans or alveolates (https://pfam.xfam.org/family/xtbd/). Some members contain RNA-binding motifs such as the double-stranded RNA binding motif (dsrm), the G-patch domain and the R3H domain, implicating their roles to recruit XRN2 to specific targets (Fig. 1B). Interestingly, yeast lacks XTBD-containing proteins, and an alternative factor Rat1-interacting protein (Rai1) binds to Rat1/XRN2 to stimulate its exoribonuclease activity (Stevens and Poole, 1995; Xiang *et al.*, 2009). Conversely, *C. elegans* DOM-3 and human DXO/DOM3Z, which are homologous with Rai1 and have decapping and pyrophosphohydrolase activities , are unlikely to bind XRN2 due to a lack of residues that are in the XRN2-binding interface of Rai1 (Xiang *et al.*, 2009; Chang *et al.*, 2012). One could speculate that coordinating Rai1’s enzymatic activities with XRN2 is advantageous for yeast, while other species chose to physically separate the two enzymes and adopted XTBD-containing proteins to expand functionality of XRN2 for their own benefit.

## XTBD family proteins

### PAXT-1

PAXT-1 was identified as a binding partner of XRN2 in *C. elegans* (Fig. 1B) (Miki *et al.*, 2014a). Localized predominantly in the nucleus, PAXT-1 stabilizes XRN2 to maintain its activity for target RNA degradation. The complex formation is particularly important at higher temperature, where animals deleted of the *paxt-1* gene cease developing and die due to loss of XRN2’s activity. *paxt-1* mutant animals can be rescued by an increased *xrn-2* gene dosage, indicating that stabilization of XRN2 is the sole essential function of PAXT-1.

### Tan1

Couvillion *et al.* identified Twi-associated novel 1 (Tan1) as a component of a ternary complex that contains XRN2 and the Argonaute/Piwi protein Twi12 in *Tetrahymena thermophila* (Fig. 1B) (Couvillion *et al.*, 2012). They found that Twi12 stabilizes XRN2, promotes its nuclear localization and stimulates its exoribonuclease activity. Consistently, disruption of the complex by Twi12 depletion reduced mature 5.8S rRNA levels and increased its precursors. In contrast, Tan1 turned out to be dispensable for activity of XRN2 and growth of *Tetrahymena*, leaving its functions in the complex unclear. In *C. elegans*, PAXT-1 is essential for development only at high temperature, where XRN2 is considerably destabilized (Miki *et al.*, 2014a; Richter *et al.*, 2016). It would be worth testing whether Tan1-deleted *Tetrahymena* grows normally in severe environments such as elevated temperature and high osmolality.

### NKRF

NKRF is a transcription factor, whose role in regulation of Interleukin-8 (IL-8) expression has been well characterized (Fig. 1B). Interaction between NKRF and NF-κB at the negative regulatory element (NRE) of the IL-8 promoter is required for repression of basal transcription and for transcriptional activation upon IL-1 stimuli (Nourbakhsh *et al.*, 2001). In the exploration of the mechanism, Rother *et al.* found that interaction of NKRF with XRN2 and negative elongation factor E (NELF-E) is required for repression of IL-8 expression in HeLa cells (Rother *et al.*, 2016). In unstimulated cells, NKRF with phosphorylated serine residues in amino acids 421 – 429 can bind to XRN2 and NELF-E to repress IL-8 transcription. Stimulation with IL-1 causes dephosphorylation of the serine residues to release XRN2 and NELF-E, leading to transcriptional activation of IL-8. How XRN2 represses IL-8 transcription remains unclear, but promoter-proximal termination of Pol II might be a possibility (Brannan *et al.*, 2012).

**Fig. 2:**
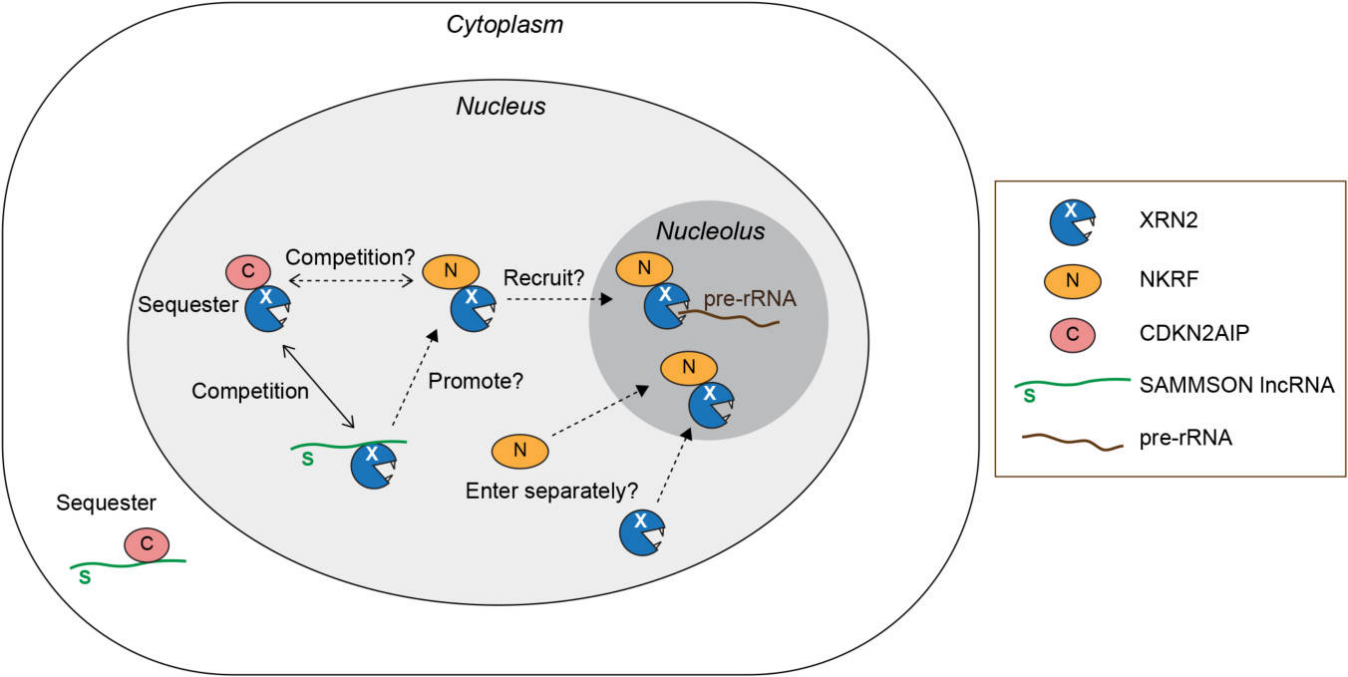
Model for XRN2 regulation by NKRF and CDKN2AIP in ribosome biogenesis. NKRF promotes localization of XRN2 to pre-rRNA for processing. It is unclear where NKRF initially recognizes XRN2, in the nucleoplasm or the nucleolus. CDKN2AIP sequesters XRN2 in the nucleoplasm, thereby suppressing pre-rRNA processing. SAMMSON promotes nucleolar localization of XRN2 by sequestering CDKN2AIP in the cytoplasm and/or by interfering with CDKN2AIP-XRN2 interaction in the nucleoplasm in melanoma cells. Competition between NKRF and CDKN2AIP for XRN2 binding and whether SAMMSON promotes NKRF-XRN2 interaction remain to be investigated.

Besides the function in the nucleoplasm, two independent studies revealed the roles of NKRF-XRN2 complexes in ribosome biogenesis in the nucleolus (Fig. 2) (Memet *et al.*, 2017; Coccia *et al.*, 2017). Memet *et al.* found that NKRF forms a complex with XRN2 and the DEAH-box RNA helicase DHX15 through direct bindings (Memet *et al.*, 2017). The complex binds to the spacer regions of pre-rRNA for processing into mature forms and elimination of excised spacer fragments. NKRF stimulates the ATPase and the helicase activities of DHX15 to promote efficient endonucleolytic cleavage at the A' site of pre-rRNA, an entry site of XRN2 for trimming, although the mechanism remains to be elucidated. Depletion of NKRF reduced the levels of XRN2 in pre-ribosomal complexes, indicating that NKRF recruits XRN2 to the complexes. Coccia *et al.* found that heat stress translocates NKRF and XRN2 from the nucleolus to the nucleoplasm, leading to accumulation of immature pre-rRNA species (Coccia *et al.*, 2017). Recovery is facilitated by HSF1 (heat shock transcription factor 1)-mediated *de novo* synthesis of NKRF, which relocalizes XRN2 to the nucleolus. XRN2 fails to relocate from the nucleoplasm to the nucleolus in NKRF-depleted cells, suggesting that NKRF is required for nucleolar localization of XRN2 during the recovery process. Thus, NKRF functions as a stress-regulated switch for ribosome biogenesis and nucleolar homeostasis by controlling subcellular localization of XRN2. It remains undetermined whether NKRF recognizes XRN2 in the nucleoplasm and recruits it to the nucleolus, or the two proteins enter the nucleolus independently, where NKRF tethers XRN2 (Fig. 2).

Two previous reports pointed out a lack of a full-length XTBD in NKRF and proposed another region to be responsible for interaction with XRN2 (Rother *et al.*, 2016; Memet *et al.*, 2017). However, these studies were apparently performed based on wrong annotations of the human NKRF mRNA sequence. A bioinformatics analysis by Alexandrova *et al.* found a sequence error in previous annotations and identified a correct NKRF mRNA with 5' extension (Alexandrova *et al.*, 2020). It translates into a 784 amino acids-long protein that contains a full-length XTBD (Fig. 1B). The team demonstrated that the NKRF protein with the full-length XTBD, but not of previous annotations, can efficiently bind to XRN2 and localize it to the nucleolus. These results support the idea that XTBD is a *bona fide* XRN2-binding domain. Perhaps another region of NKRF plays a minor role to reinforce the binding mediated by XTBD.

## CDKN2AIP

CDKN2AIP, also known as CARF (Collaborator of ARF), regulates cell proliferation by stabilizing the tumor suppressor p53 (Fig. 1B) (Hasan *et al.*, 2002, 2004; Wadhwa *et al.*, 2017). Sato *et al.* identified XRN2 as a direct binding partner of CDKN2AIP in human cells and examined their roles in pre-rRNA processing (Sato *et al.*, 2015). Knockdown of CDKN2AIP shifted the balance of XRN2 localization from the nucleoplasm to the nucleolus, while its overexpression had the opposite effect, leading to accumulation of 5'-extended forms of pre-rRNA. These findings suggest that CDKN2AIP sequesters XRN2 in the nucleoplasm to prevent it from processing pre-rRNA in the nucleolus (Fig. 2). Thus, CDKN2AIP apparently opposes the function of NKRF that promotes nucleolar localization of XRN2. Since an XTBD allows only one XRN2 molecule to bind (Richter *et al.*, 2016), NKRF and CDKN2AIP form distinct complexes with XRN2 in a same cell (Miki *et al.*, 2014a). An intriguing question for future investigation is weather the two proteins competitively control nucleolar accumulation of XRN2 and hence ribosome biogenesis (Fig. 2).

A long non-coding RNA (lncRNA) SAMMSON emerged as another player in this regulation. It was originally identified as a melanoma-specific lncRNA that promotes maturation of mitochondrial 16S rRNA through interaction with complement C1q binding protein (C1QBP) and supports cell survival (Leucci *et al.*, 2016). A follow-up study by the team identified CDKN2AIP and XRN2 as interacting partners of SAMMSON and demonstrated that SAMMSON disrupts CDKN2AIP-XRN2 interaction to promote nucleolar localization of XRN2, leading to elevated levels of 18S rRNA (Vendramin *et al.*, 2018). Given that SAMMSON and CDKN2AIP bind to overlapping regions of XRN2, the researchers proposed that SAMMSON interferes with the formation of CDKN2AIP-XRN2 complexes by two distinct mechanisms: masking the CDKN2AIP-binding region of XRN2 and tethering CDKN2AIP to the cytoplasm (Fig. 2). Thus, SAMMSON promotes biogenesis of both mitochondrial and cytoplasmic ribosomes, thereby protein synthesis, which is required for the growth and survival of melanoma cells.

## CDKN2AIPNL

CDKN2A interacting protein N-terminal like (CDKN2AIPNL) is a small protein with an XTBD as a sole annotated domain (Fig. 1B). Although its cellular function remains uncharacterized, Richter *et al.* demonstrated that CDKN2AIPNL and XRN2 can form a complex in humans (Richter *et al.*, 2016). Interestingly, human CDKN2AIPNL was able to functionally substitute for PAXT-1 in *C. elegans* to stabilize XRN2 and support survival of the animals. Whether CDKN2AIPNL stabilizes XRN2 in human cells remains to be investigated, while depletion of neither NKRF nor CDKN2AIP reduced XRN2 levels (Sato *et al.*, 2015; Memet *et al.*, 2017).

## Perspective

Although XRN2 is an essential gene (Amberg *et al.*, 1992; Miki *et al.*, 2014b), its roles in growth or development have been unexplained by its cellular functions. This is largely due to the multifunctional nature of XRN2, whose loss results in pleiotropic defects (Miki *et al.*, 2014b). Functional and phenotypic characterization of XTBD family proteins may help to address the issue by separating a specific function of XRN2 from the rest. Conversely, the finding of XTBD, which can link functionalities of a protein to XRN2, may shed light on unexplored family members. For example, the *Drosophila melanogaster* protein CG31301, which contains XTBD, binds to siRNAs and promotes RNAi by an unknown mechanism (Fig. 1B) (Gerbasi *et al.*, 2010). It would be worth investigating whether XRN2 is involved in degradation of target mRNA in this process. Another example of interest is a group of proteins with two XTBDs such as *Danio rerio* CDKN2AIP (Fig. 1B). Given that this domain architecture is found exclusively in fish, exploring the functions of these proteins may open a new window for understanding how XRN2 contributes to creating the characteristics of the species.

## Abbreviations

Rat1: ribonucleic acid trafficking protein 1
*C. elegans*: *Caenorhabditis elegans*
XTBD: XRN2-binding domain
Pol II: RNA polymerase II
PAXT-1: Partner of XRN-Two 1
NKRF: NF-κB repressing factor
CDKN2AIP: CDKN2A interacting protein
dsrm: double-stranded RNA binding motif
Rai1: Rat1-interacting protein
Tan1: Twi-associated novel 1
IL: Interleukin
NELF-E: negative elongation factor E
CDKN2AIPNL: CDKN2A interacting protein N-terminal like
